# Genetically predicted levels of circulating cytokines and the risk of six immune skin diseases: a two-sample Mendelian randomization study

**DOI:** 10.3389/fimmu.2023.1240714

**Published:** 2023-10-26

**Authors:** Qinghua Luo, Qiurui Cao, Jinyan Guo, Shuangqing Chang, Yunxiang Wu

**Affiliations:** ^1^ Department of Anorectal Surgery, Jiangmen Wuyi Hospital of Traditional Chinese Medicine, Jiangmen, China; ^2^ Department of Anorectal Surgery, Affiliated Hospital of Jiangxi University of Chinese Medicine, Nanchang, China

**Keywords:** Mendelian randomization, circulating cytokines, immune skin diseases, genome-wide association studies, causal relationship

## Abstract

**Background:**

Circulating cytokines play a crucial role in the onset and progression of immune skin diseases. However, the causal relationships and the direction of causal effects require further investigation.

**Methods:**

Two-sample Mendelian randomization (MR) analyses were conducted to assess the causal relationships between 41 circulating cytokines and six immune skin diseases including alopecia areata, chloasma, hidradenitis suppurativa (HS), lichen planus (LP), seborrheic dermatitis, and urticaria, using summary statistics from genome-wide association studies. Reverse MR analyses was performed to test for the reverse causation. Pleiotropy and heterogeneity tests were conducted to assess the robustness of the findings.

**Results:**

Twelve unique cytokines showed a suggestive causal relationship with the risk of six immune skin diseases. Among them, the causal effects between 9 unique cytokines and immune skin diseases have strong statistical power. Additionally, the concentrations of six cytokines might be influenced by LP and urticaria. After Bonferroni correction, the following associations remained significant: the causal effect of beta-nerve growth factor on HS (odds ratio [OR] = 1.634, 95% confidence interval [CI] = 1.226-2.177, p = 7.97e-04), interleukin (IL)-6 on LP (OR = 0.615, 95% CI = 0.481-0.786, p = 1.04e-04), IL-4 on LP (OR = 1.099. 95% CI = 1.020-1.184, p = 1.26e-02), and IL-2 on urticaria (OR = 0.712, 95% CI = 0.531-0.955, p = 2.33e-02).

**Conclusion:**

This study provides novel perspectives on the relationship between circulating cytokines and immune skin diseases, potentially providing valuable insights into their etiology, diagnostic approaches, and treatment.

## 1 Introduction

The skin, being the body’s largest immune organ, serves as a protective barrier against external threats. Numerous skin diseases are associated with the overall homeostasis of the body, including inflammatory response, immune status, metabolic level, and gut flora homeostasis (1, 2). Immune skin diseases, such as alopecia areata (AA), chloasma, hidradenitis suppurativa (HS), lichen planus (LP), seborrheic dermatitis (SD), and urticaria, are highly prevalent and contribute significantly to the global health burden. However, due to their complex and diverse pathogenesis, effective treatments for these conditions are limited (3–8). Genetic research, particularly through genome-wide association studies (GWAS), has identified several single-nucleotide polymorphisms (SNPs) associated with skin diseases (9). However, translating these findings into clinically relevant therapeutic targets and treatments still requires substantial efforts.

Skin diseases often exhibit systemic inflammatory changes (10, 11). Circulating cytokine levels, as crucial inflammatory regulators, exert a significant influence on immune and inflammatory responses *in vivo* (12). Over 300 cytokines have been identified, primarily including chemokines, interleukins (ILs), interferons (IFNs), and growth factors. These small soluble molecules, secreted by cells, play roles in differentiation, proliferation, and apoptosis promotion (13). Disturbances in cytokine expression are a prominent feature during skin disease episodes (14). During exacerbations, patients with AA show increased levels of serum pro-inflammatory factors such as IFN-γ, IL-2, IL-7, and IL-15 (15). Furthermore, there have been reports on the association between chloasma, HS, LP, SD, and urticaria and cytokines (16–20). However, the existing evidence linking cytokines to these immune skin diseases stems from cross-sectional studies, which are susceptible to confounding factors, such as environment, age, dietary preferences, and lifestyles. Therefore, the causal association between cytokines and these immune skin diseases, as well as the direction of the causal effect remain unknown.

Mendelian randomization (MR) is currently a popular means for exploring causal relationships between exposures and outcomes of interest (21). It leverages the fundamental principles of Mendel’s law, which entail the “random assignment of parental alleles to offspring”, resembling the random assignment of participants in a randomized controlled trial. Thus, MR has emerged as a valuable tool for exploring causal relationships between complex traits, diseases, and phenotypes (22).

In the present study, genetic variants associated with 41 circulating cytokines were obtained from a large GWAS (23). Furthermore, a bidirectional two-sample MR analysis was conducted to investigate the relationship between cytokines and six immune skin diseases. Our findings might help elucidate the association between systemic inflammation and these immune skin diseases, offering new diagnostic biomarkers and revealing new targets for drug therapy.

## 2 Materials and methods

### 2.1 Study design

**Figure 1:**
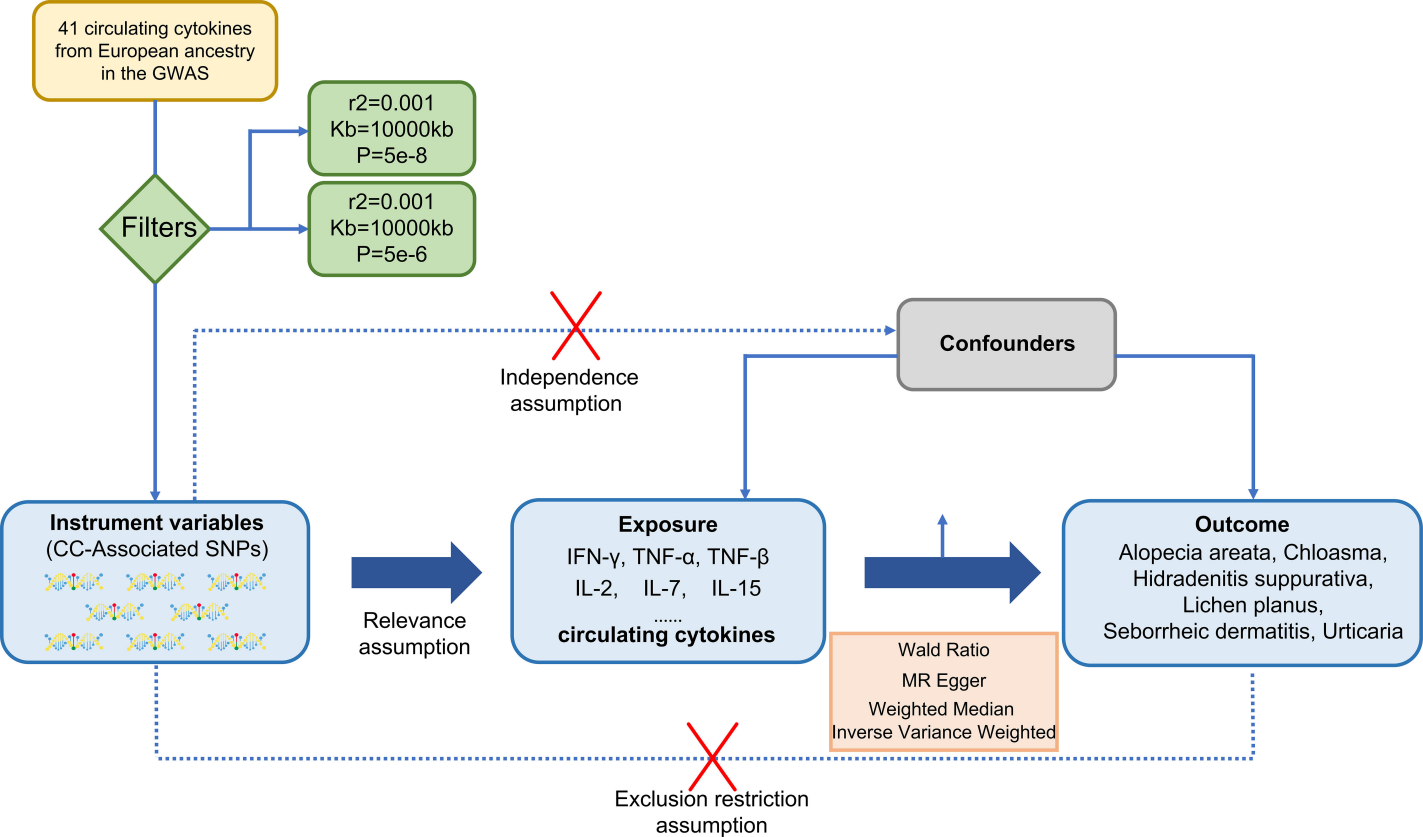
A design of three basic assumptions of Mendelian-randomization analysis: (1) a strong correlation between SNPs and exposure; (2) absence of association between SNPs and confounding factors that might influence the association between exposure and outcome; (3) exposure being the only pathway through which SNPs exert an effect on the outcome. CC, circulating cytokines; IFN-γ, Interferon-gamma; TNF-α, Tumor necrosis factor-alpha; TNFβ, Tumor necrosis factor-beta; IL-2, Interleukin-2; IL-7, Interleukin-7; IL-15, Interleukin-15.

In the MR analysis, SNPs were treated as valid instrumental variables (IVs). These IVs had to fulfil three key assumptions, as shown in **Figure 1**: (1) a strong correlation between SNPs and exposure; (2) absence of association between SNPs and confounding factors that might influence the association between exposure and outcome; (3) exposure being the only pathway through which SNPs exert an effect on the outcome (24).

### 2.2 Data sources

The GWAS conducted by Ahola-Olli et al. provided the genome-wide association summary statistics for 41 systemic inflammatory regulators (23). The study population comprised 8,293 individuals from three different Finnish cohorts, namely the FINRISK1997, FINRISK2002, and the Young Finns Cardiovascular Risk Study (YFS) (**Supplementary Table S1**). Cytokines and growth factors in plasma were measured for participants in the FINRISK1997 cohort, while heparin plasma and serum cytokine measurements were performed for individuals in the FINRISK2002 and YFS cohorts. The circulating cytokine levels were expressed as standard deviations. Genetic associations were adjusted for the first ten genetic principal components, age, sex, and body mass index. Complete GWAS summary statistics were obtained from https://www.ebi.ac.uk/gwas/downloads/summary-statistics.

The summary statistics for the six immune skin diseases were obtained from FinnGen GWAS available at https://r8.finngen.fi/ (25). The numbers of cases and controls for each phenotype was as follows: 682 patients with AA and 361,140 controls, 95 patients with chloasma and 329,443 controls, 931 patients with HS and 361,140 controls, 3,597 patients with LP and 364,071 controls, 2,688 patients with SD and 336,589 controls, and 3,495 patients with urticaria and 364,583 controls.(More details as) These skin diseases were defined based on the International Classification of Diseases, 10^th^ revision.

### 2.3 Selection of IVs

A genome-wide statistical significance threshold of <5.0 × 10^-8^ was established to extract the SNPs robustly associated with the studied exposures. Additionally, the r^2^ threshold of 0.001 and a clump window size of 10,000 kb were set to eliminate linkage disequilibrium (26). As a result, 27 cytokines were retained based on these criteria. In a secondary analysis, the genome-wide significance threshold was relaxed to 5.0 × 10^-6^ to allow for the inclusion of all 41 cytokines.

For the reverse direction, the significance threshold employed was p <5 × 10^-8^, with consistent clumping parameter with the forward-direction analysis. However, due to the lack of sufficient SNPs, four diseases, namely AA, chloasma, HS, and SD, were not subjected to the reverse MR analysis. Consequently, the revere MR analyses were only conducted for LP and urticaria.

### 2.4 Statistical analysis

The Wald ratio test was used to estimate the association between identified IVs and skin diseases for cases containing only one SNP (27). For multiple SNPs, three MR analysis methods were used, including inverse variance weighted (IVW), MR-Egger and weighted median (WM), with the IVW method being the predominant method. The IVW method, which is the classical approach for MR analysis, incorporates inverse variances of each IV as weights to calculate a weighted average while ensuring the validity of all IVs (28). MR-Egger, on the other hand, employs a form of weighted linear regression analysis. Estimates derived from this method are robust and independent of IV validity, although they might have lower statistical precision and are susceptible to outlying genetic variations (29). The WM method addresses the issue of a large variation in estimation precision. Similar to the IVW method, this approach assigns inverse weights based on the variance of each genetic variant, and it demonstrates reliability even in the presence of violated causal effects (30). When significant causal correlations are evaluated using the IVW method, the MR egger and WM methods serve as supplementary methods and directional validation.

MR-Egger regression was used to assess pleiotropy. Moreover, the presence of pleiotropy and identification of outlying SNPs could be determined through the MR-Pleiotropy RESidual Sum and Outlier (PRESSO) test (31). The Cochran’s Q was used to assess heterogeneity. A p-value of <0.05 indicates the presence of heterogeneity.

The strength of IVs can be evaluated using the F- statistic, which is calculated as follows: F = β^2^/SE^2^, where β is the effect size of the allele and SE is the standard error (32). An F statistic greater than 10 suggests the absence of weak IV bias (33). When analyzing multiple outcomes, significance thresholds are adjusted using the Bonferroni method. A p value smaller than the Bonferroni-corrected threshold indicates statistical significance. Additionally, correlations not exceeding the Bonferroni-corrected significance level but <0.05 were considered suggestively significant. All analyses were performed using R version 4.2.2, with the software packages “TwoSampleMR” (34) and “MRPRESSO” (28).

Due to the limited variability of phenotypic characteristics and the small proportion of patients in the outcome trait GWAS data, statistical ability is crucial. In order to calculate the statistical power, an online tool called mRnd (35) was used. In the case of a Type I error of 5%, we determined the expected OR threshold for each circulating cytokine and immune skin disease, which should meet a minimum power requirement of>80%.

## 3 Results

### 3.1 Causal effects of circulating cytokines on six immune skin diseases

**Figure 2:**
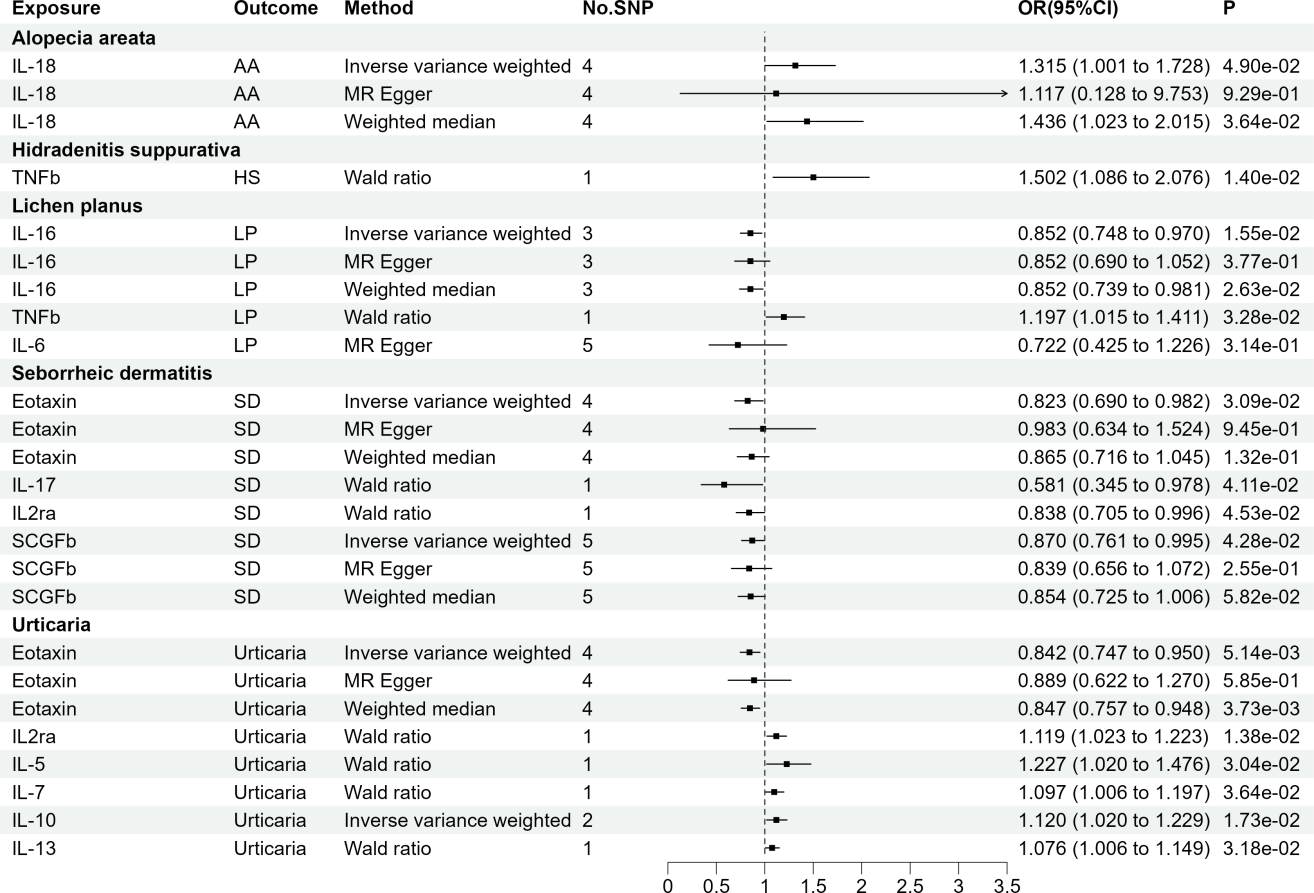
The 14 causal relationships (SNPs reaching P<5×10^-8^). AA, Alopecia areata; HS, Hidradenitis suppurativa; LP, Lichen planus; SD, Seborrheic dermatitis; IL-18, Interleukin-18; TNFβ, Tumor necrosis factor-beta; IL-16, Interleukin-16; IL-17, Interleukin-17; IL2rα, Interleukin-2 receptor, alpha subunit; SCGFβ, Stem cell growth factor beta.

**Figure 2** illustrates the results, with 14 sets of suggestively significant associations (p <0.05) identified at a significance threshold of <5.0 × 10^-8^. Based on the significant correlation between circulating cytokines and immune skin diseases assessed by Mendelian randomization, further statistical efficacy analysis examined the threshold range of OR that needs to be reached when meeting sufficient statistical efficacy. It is worth noting that six cytokines meet the OR threshold for statistical efficacy analysis (IL-18, IL-17, IL-16, IL2α, TNFβ, Eotaxin). The details of statistic power analyses are presented in **Supplementary Table 6**. The above results do not meet the threshold range corrected by Bonferroni (p = 0.05/27 = 1.85e-03). The specific SNP profiles, as well as the pleiotropy and heterogeneity results of these 14 groups, are presented in **Supplementary Tables S3**, **4**. No evidence was observed in terms of heterogeneity and pleiotropy (p >0.05). The F-statistics for all groups were >10, indicating the absence of weak IV bias. **Supplementary Table S5** presents the MR analyses involving 27 cytokines and six immune skin diseases.

**Figure 3:**
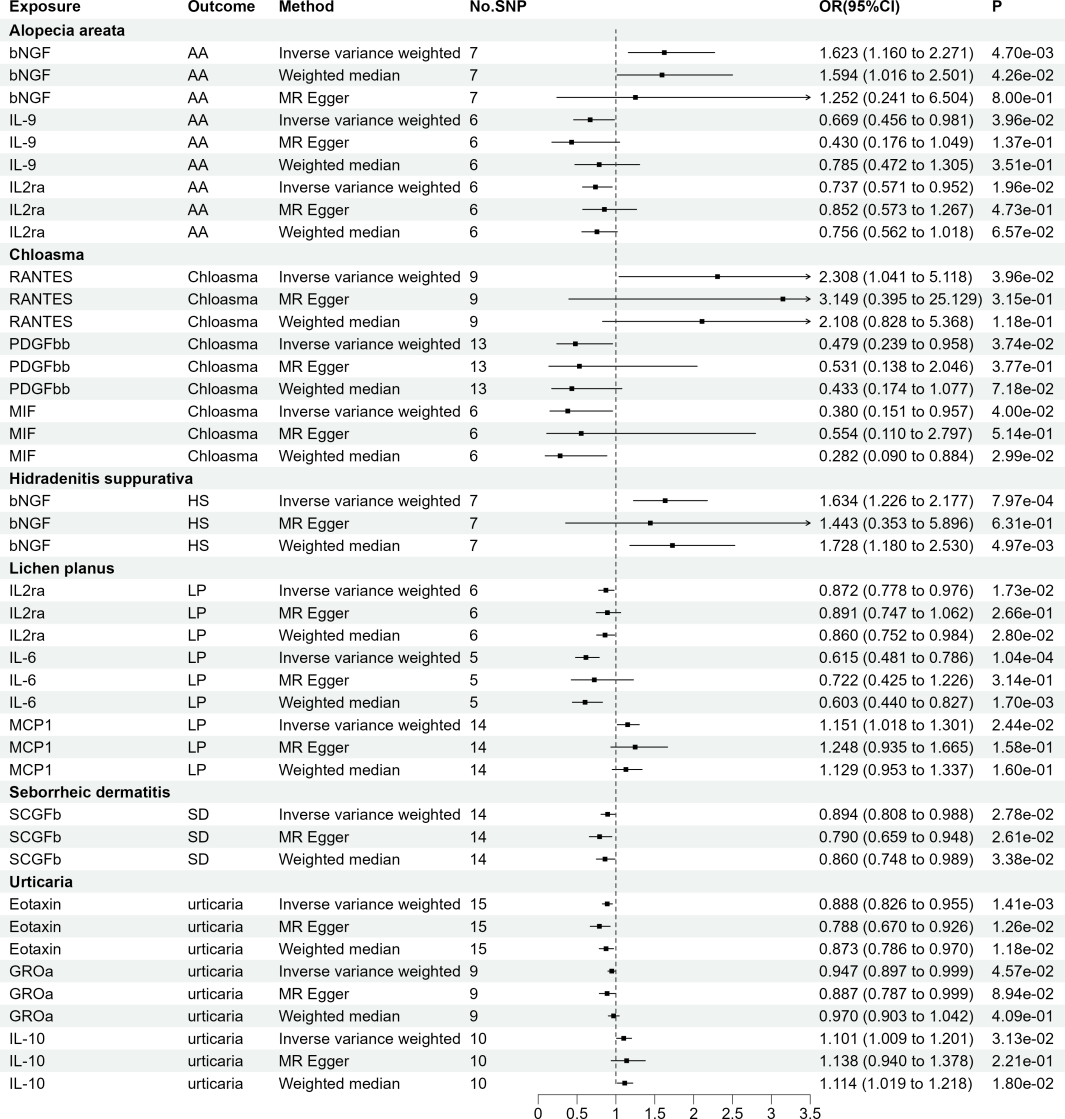
The 14 causal relationships (SNPs reaching P<5×10^-6^). AA: Alopecia areata, HS, Hidradenitis suppurativa; LP, Lichen planus; SD, Seborrheic dermatitis; βNGF, Beta nerve growth factor; IL-9, Interleukin-9; IL2rα, Interleukin-2 receptor; MIF, Macrophage migration inhibitory factor; PDGFbb, Platelet derived growth factor BB; RANTES, Regulated on Activation, Normal T Cell Expressed and Secreted; IL-6, Interleukin-6; MCP1, Monocyte chemotactic protein-1; SCGFβ, Stem cell growth factor beta; GROα, Growth regulated oncogene-α; IL-10, Interleukin-10.

As presented in **Figure 3**, employing a significance threshold of <5.0 × 10^-6^, 14 sets of suggestively significant correlations (p <0.05) were obtained. Based on the significant correlation between circulating cytokines and immune skin diseases assessed by Mendelian randomization, further statistical power analysis examined the threshold range of OR that needs to be reached when meeting sufficient statistical efficacy. Among the 14 pairs of suggestive significantly correlated traits, 4 pairs of suggestive significantly correlated traits that meet sufficient statistical efficacy were obtained through screening. The details of statistic power analyses are presented in **Supplementary Table 10**. Following Bonferroni correction (p = 0.05/41 = 1.22e-03), bNGF remained statistically significant in HS (OR = 1.634, 95% CI = 1.226-2.177, p = 7.97e-04) and IL6 remained statistically significant in LP (OR = 0.615, 95% CI = 0.481-0.786, p = 1.04e-04). **Supplementary Tables S7**, **8** provide detailed information on the SNP profiles, as well as the details of pleiotropy and heterogeneity. No heterogeneity or pleiotropy was observed, and there was no evidence of weak IV bias. The details of MR analyses involving 41 cytokines and six immune skin diseases are presented in **Supplementary Table S9**.

### 3.2 Causal effects of six immune skin diseases on circulating cytokines

**Figure 4:**
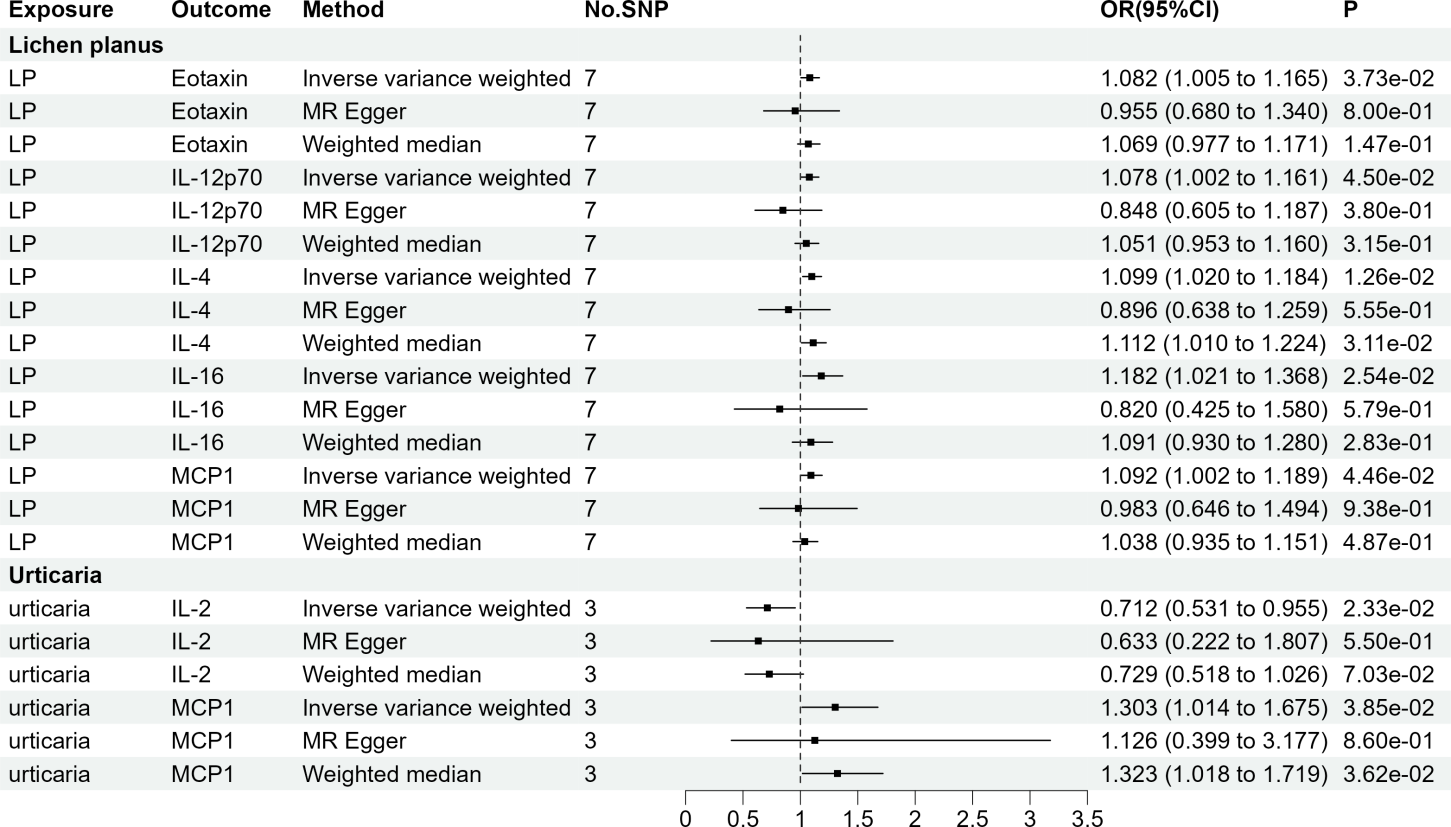
The 7 causal relationships (SNPs reaching P<5×10^-8^). LP, Lichen planus; IL-12p70, Interleukin-12p70; IL4, Interleukin-4; IL16, Interleukin-16; MCP1, Monocyte chemotactic protein-1; IL2, Interleukin-2.

As AA, chloasma, HS, and SD did not have genome-wide significant SNPs, our assessment was limited to LP and urticaria. Finally, seven sets of suggestively significant correlations (p <0.05) involving six cytokines (eotaxin, IL-12p70, IL-2, IL-4, IL-16, and MCP-1) were obtained. The association of LP with IL-4 (OR = 1.099, 95% CI = 1.020-1.184, p = 1.26e-02) and urticaria with IL-2 (OR = 0.712, 95% CI = 0.531-0.955, p = 2.33e-02) remained significant at the Bonferroni-corrected significance threshold (p = 0.05/2 = 0.025). **Figure 4** shows the Mendelian randomization randomized forest map results of circulating cytokines and immune skin diseases with statistically significant (P<0.05) IVW method using multiple methods. **Supplementary Tables S9**, **10** provide information on SNPs, heterogeneity, and pleiotropy for these groups. Moreover, the F-statistics indicate the absence of weak IV bias. Detailed results of the specific reverse MR analyses involving LP and urticaria are presented in **Supplementary Table S11**.

**Figure 5:**
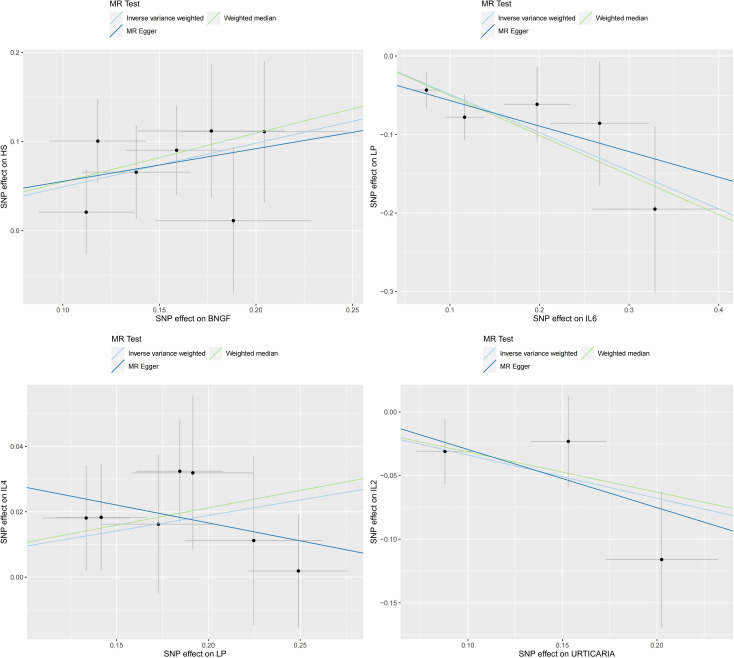
Scatter plots for the 4 significant associations at the Bonferroni corrected significance threshold. β NGF: Beta nerve growth factor, HS, Hidradenitis suppurativa; IL2, Interleukin-2; IL4, Interleukin-4; IL6, Interleukin-6; LP, Lichen planus; SD, Seborrheic dermatitis.

The four groups that remained statistically significant after Bonferroni correction in this bidirectional MR analysis were selected for scatter plotting, as presented in **Figure 5**.

## 4 Discussion

This is the first study performing a bidirectional MR analysis to explore the relationship between 41 circulating cytokines and six immune skin diseases (AA, chloasma, HS, LP, SD, and urticaria). When cytokines were considered the exposure variable (p <5.0 × 10^-6^), potential associations were observed between 3 cytokines and the risk of immune skin diseases. Specifically, bNGF was associated with AA and HS, and RANTES was associated with chloasma, IL-6 was associated with LP. On the other hand, when immune skin diseases served as the exposure variable, LP affected eotaxin, IL-12p70, IL-4, IL-16, and MCP-1 and urticaria affected IL-2 and MCP-1. Due to the limited number of cases available in the FinnGen database for the remaining four skin diseases, the genetic variation was insufficient for effective exploration. Therefore, a reanalysis should be performed in the future when an adequate number of patients become available. Although there are some circulating cytokines that have causal associations with immune skin diseases that have not been analyzed through statistical efficacy, some of these cytokines may have predictive value for disease risk and diagnostic purposes. Considering the complexity and complexity of the cytokine network in the body, it is necessary to conduct further clinical and basic experiments to verify these hypotheses.

Healthy skin comprises three layers, namely the epidermis, dermis, and subcutaneous fat. These layers harbor resident cell populations, both immune and non-immune cells, which play crucial roles in maintaining a strong barrier against foreign substances. Moreover, these cells are involved in various inflammatory processes, contributing to an immune response against infections, autoimmunity, tumor immunity, and allergies (10). It can be stated that the pathogenesis of skin diseases is, to a certain extent, linked to inflammatory responses.

AA, a prevalent autoimmune disease, is characterized by non-scarring alopecia. The pathological process of AA involves a shift in the hair follicle microenvironment from a state of immune homeostasis to an active inflammatory state (15). Several GWASs have identified IL2ra as a cytokine gene associated with AA (36–38). IL2ra functions through regulatory T cells, playing a crucial role in maintaining immune homeostasis and suppressing autoantigen-mediated immune responses (39, 40). Notably, the *in vivo* IL-9 levels are significantly decreased in patients who exhibit effective responses to tetraphenyl cyclopentadienone treatment (41). These findings align with the results obtained in the current study. At present, no research has revealed the biological association and mechanism between bNGF and AA. Our study suggests for the first time that bNGF may have the potential to become an early screening biomarker for AA in the future and provide assistance in exploring the biological mechanisms and therapeutic targets of AA.

Chloasma, a prevalent light-induced pigmentation disorder, results in skin inflammation and the release of melanogenic cytokines and growth factors in response to ultraviolet (UV) radiation. These factors play a crucial role in the hyperpigmentation and reactivation of chloasma lesions (42). MIF is considered a key player in skin biology and wound healing and is negatively correlated with cellular and tissue ageing (43). MIF might serve as a key mediator in photoaging. *In vivo* and *in vitro* studies have shown that UVB radiation (UVB) induces the expression of MIF messenger ribonucleic acid in the skin and keratinocyte MIF production *in vivo* and *in vitro* (44). Moreover, UVB has been found to inhibit skin inflammation by inhibiting the production of RANTES in epidermal keratinocytes. This provides a new direction for the treatment of skin inflammation (45). PDGFbb production is reduced in terms of quantity and activity by UVB radiation (46). In addition, it promotes wound healing (47). Furthermore, UV irradiation induces RANTES overgrowth, resulting in inflammation (45).

HS, a chronic and inflammatory skin disease, imposes a significant burden on individuals and predominantly affects the axillae, groin, buttocks, and perianal areas. It is characterized by abnormal activation of the innate immune system (48, 49). While specific studies exploring the association between HS and bNGF are currently lacking, the observed relationship between the two cytokines remains significant even after correction. Moreover, a recent study on the relationship between bNGF and chloasma reported higher bNGF levels in affected areas compared with the normal sites (50). Therefore, the relationship between HS and bNGF warrants further investigation.

LP is a T cell-mediated autoimmune disease, specifically CD8^+^ T cells, which are recruited into the skin and contribute to interface dermatitis (51). Impairment of IL2ra function in patients with oral LP (OLP) results in an increased inflammatory response (52). MCP-1, a chemokine present in LP lesions, plays a crucial role in aggregating monocytes to the skin interface and inducing toxic effects (53). LP and MCP-1 exhibit a bidirectional causal association that warrants further investigation. Reverse MR studies have demonstrated elevated cytokine IL-4 levels in the blood and saliva of patients with OLP (54). The relationship between LP and eotaxin, IL-12p70, and IL163 is novel and requires further investigation.

SD is a chronic, recurrent inflammatory skin disease. SCGFb is a newly discovered protein-secreting sulphated glycoprotein that serves as a growth factor in early hematopoiesis (55). Study have shown that SCGFb may be able to inhibit the pathogenesis of proliferative diabetic retinopathy(PDR) (55). However, the underlying mechanism remains to be elucidated.

Urticaria is a skin disease characterized by clinical symptoms such as wheal and angioedema. The pathogenesis of urticaria involves the activation of mast cells, which in turn release multiple cytokines, leading to sensory nerve activation, vasodilation, and plasma extravasation (56). Chronic urticaria is characterized by increased eotaxin and MCP-1, which are indicative of an inflammatory response (57, 58). Urticaria is mediated by mixed T helper (Th) 1/Th2-reactive lymphocytes, with Th1 cells primarily secreting IL-2 and Th2 cells secreting IL-10. In patients with urticaria, there is an observed increase in IL-10 production and a decrease in IL-2 levels, consistent with the findings of the present study (59).

The present study has certain limitations. Our study elucidates the intricate relationship between circulating cytokines and the risk of six immune skin diseases. However, it’s imperative to recognize its limitations, which warrant attention in subsequent research. Despite our meticulous selection of instrumental variables (IVs) and the deployment of statistical tests, such as MR-PRESSO and MR-Egger intercept, to address potential pleiotropy and confounding, these concerns are not entirely eliminable. Future endeavors should place greater emphasis on understanding and rectifying these challenges, bolstering the validity of conclusions drawn from such data. Moreover, the representativeness of our results is constrained by the limited sample sizes for specific diseases and inflammatory markers. Although F-statistics indicate the potential insignificance of weak IVs, the precision of our inferences, particularly for these diseases and markers, remains ambiguous.

The primary scope of our findings pertains to European populations, given the Finnish origin of our dataset. The mosaic of genetic variations across ethnicities and geographies underscores the need for more comprehensive studies. Genetic disparities have profound implications in clinical scenarios, necessitating the analysis of more heterogeneous GWAS data in the future. Of the 41 cytokines we scrutinized, a mere six demonstrated a pronounced association with skin immune diseases. This underscores their specialized role in disease pathogenesis. However, the magnitude of these associations can be contingent upon factors like sample size, genetic heterogeneity, and disease-specific nuances. The cellular dynamics within skin immune diseases demand further exploration. Deciphering the interactions between various skin cell populations and their associated cytokines will enrich our understanding of disease etiology. Cutting-edge techniques, such as single-cell RNA sequencing and spatial transcriptomics, promise to be revelatory in this domain.

While our Mendelian stochastic approach was instrumental in inferring causality, clinical implications necessitate a more direct approach. This entails measuring cytokine levels in both the affected and healthy populace, juxtaposing them with observed gene polymorphisms. Longitudinal assessments, which monitor the influence of these polymorphisms on cytokine levels across diverse conditions, are paramount.

In conclusion, our study provides a preliminary framework on the cytokine dynamics in immune skin diseases. To fully harness its translational potential, it’s pivotal to address the aforementioned limitations, broaden the scope of datasets, and employ sophisticated methodologies. The potential biomarkers identified warrant rigorous validation in expansive, diverse cohorts and systematic clinical evaluations before they can be integrated into clinical practice guidelines.

## 5 Conclusion

In summary, this bidirectional MR analysis indicates potential causal associations between specific circulating cytokines and the six immune skin diseases. These findings coffer insights into potential targets and novel biomarkers for their diagnosis and treatment of immune skin diseases in clinical settings.

## Data availability statement

Publicly available datasets were analyzed in this study. This data can be found here: Data for 41 circulating cytokines are available at https://www.ebi.ac.uk/gwas/downloads/summary-statistics. Data for the six immune skin diseases can be downloaded from https://r8.finngen.fi.

## Ethics statement

Ethical review and approval were not required for the study on human participants in accordance with the local legislation and institutional requirements. Written informed consent from the participants’ legal guardian/next of kin was not required to participate in this study in accordance with the national legislation and the institutional requirements.

## Author contributions

QL: Conceptualization, methodology, formal analysis, data curation, writing-original draft preparation; QC: Data curation, writing-original draft preparation, visualization; JG: Writing-original draft preparation, visualization; SC: Data curation, visualization; YW: Supervision, writing-review and editing. All authors contributed to the article and approved the submitted version.
